# Retrospective comparison of functional and radiological outcome, between two contemporary high flexion knee designs

**DOI:** 10.1051/sicotj/2016026

**Published:** 2016-10-18

**Authors:** Vikash Kapoor, Daipayan Chatterjee, Sutanu Hazra, Anirban Chatterjee, Parag Garg, Kaustav Debnath, Soham Mandal, Sudipto Sarkar

**Affiliations:** 1 Medica Institute of Orthopaedics, Medica Superspeciality Hospital Mukundapur Kolkata 700099 West Bengal India

## Abstract

*Introduction*: Patient satisfaction after total knee replacement (TKR) depends on the amount of pain relief and the functional activities achieved. An important criterion of good functional outcome is the amount of flexion achieved and whether the patient can manage high flexion activities. In order to increase the amount of safe flexion, various implant designs have been developed. This study aims to compare the outcome after TKR using two contemporary high flexion knee designs: Sigma CR150 High Flex Knee prosthesis (Depuy, Warsaw, Indiana) and NexGen High Flex Knee prosthesis (Zimmer, Warsaw, Indiana).

*Material*: A retrospective study was conducted with 100 cases of each design and their functional and radiological outcome was assessed after two years of follow-up.

*Results*: The two groups had comparable results in terms of subjective satisfaction, range of motion achieved and radiological outcome. Depuy group fared better than Zimmer in terms of functional outcome (modified Oxford knee score).

*Conclusion*: Depuy group was found to have fared better than Zimmer in terms of functional outcome. However, it is very difficult to rate one design above the other based on our small sample size and short duration of follow-up.

## Introduction

Patient satisfaction after total knee replacement (TKR) depends on the amount of pain relief and the functional activities achieved. An important criterion of good functional outcome is the amount of flexion achieved and whether the patient can manage high flexion activities such as crouching, kneeling and getting out of low chair [1]. In order to increase the amount of safe flexion, various implant designs have been designed. There have been studies comparing normal flexion and high flexion designs of implants of the same company [2, 3]. However, there has been limited research on the efficacy of the different high flexion designs commonly available. This study aims to compare the outcome after TKR using two contemporary high flexion knee designs with fixed bearing tibial base plate: Sigma CR150 High Flex Knee prosthesis (Depuy, Warsaw, Indiana) and NexGen High Flex Knee prosthesis (Zimmer, Warsaw, Indiana).

## Methods

A retrospective study was conducted on cases with primary TKR done by the senior author using any of the two previously mentioned implant designs which had at least two years of follow-up.

Patients were excluded if they had:

1. inflammatory or secondary osteoarthritis (OA) of knee;
2. severe varus or valgus deformity (>30°);
3. bone loss requiring tibial or femoral augments;
4. disorders of hip, foot, ankle or spine which limit mobility;
5. disorders of central nervous system such as dementia, parkinsonism and other severe co-morbidities including morbid obesity which hamper mobility.

Out of 1400 TKRs done by the senior author, 218 patients met our selection criteria (115 with Depuy implant and 103 with Zimmer implant). However, for the ease of calculation we randomly selected 100 from each group by a card selection method. The implant used was based on patient’s informed choice of the same and consent for surgery. Our Institutional Review Board granted ethical approval and all participants gave written consent to participate in the study.

### Surgical technique

The senior author performed all the TKRs. The procedure was performed through a midline skin incision with a medial para-patellar approach with no difference in soft-tissue dissection between the two groups. The anterior cruciate ligament was excised while the posterior cruciate ligament was retained in all the knees. In both groups, femoral preparation was done first followed by tibial preparation. Resection of the distal femur was done to remove a thickness of bone equal to that of the femoral component to be implanted. Tibial cut was taken to resect the minimum thickness of bone needed for soft-tissue balancing, leaving a surface that was perpendicular to the shaft of the tibia in the coronal plane with a 7° posterior slope in the sagittal plane. In resection of the femur and tibia, care was taken to balance the flexion and extension gaps and to alleviate any flexion contracture. Patella was not resurfaced. Tourniquet was used just before cementing and released after compression dressing was applied. No drain was inserted. Patients were started on physiotherapy for muscle strengthening and knee bending from the next day. As patients received epidural infusion post surgery for three days for pain relief, full weight bearing walking was allowed from day one post surgery with walker support and a long knee brace. The long knee brace was removed during knee bending exercises. Stair climbing and commode training were started on day two. Patients were discharged on day three and home-based physiotherapy by hospital physiotherapist was continued for three weeks. The long knee brace was removed after gaining adequate quadriceps muscle strength so as to prevent buckling of the knee while walking (approximately two weeks). The walker was continued for one week followed by cane walking for another one week followed by unassisted weight bearing after two weeks.

### Patient evaluation

Pre-operative and two years post-operative clinical, functional and radiological data were retrieved from our hospital database for evaluation and analysis. Clinical and functional assessment was done using revised Oxford knee scoring system [4] and the Western Ontario and McMaster Universities Arthritis Index (WOMAC) scoring system [5]. Radiographs done before and after surgery included antero-posterior views both standing and supine, a lateral film and a skyline patellar view with 90° of flexion of the knee. Both pre- and post-operative scoring, range of motion (ROM) measurement (using goniometer) and radiographic evaluation were done by two blinded observers who were not part of the operating team and who did not know the type of implant received by the patient. Any detectable osteolysis around the components was recorded along with assessment of knee alignment, position of the components and subluxation or dislocation of patella.

## Results

Depuy CR 150 system was used in 100 knees and Zimmer High Flex in the other 100. Follow-up was at least two years (range: 24–32 months). Pre-operative findings have been compiled in Table 1. Intra-operative details such as implant sizes used are enumerated in Table 2. None of the patients had any intra-operative life-threatening or implant-related complication. Five patients (four from Zimmer and one from Depuy group) had a problem in the healing of the suture line primarily. They required a single debridement and re-suturing after three weeks of surgery and the wound healed subsequently. None had any episode of infection, peri-prosthetic fracture or implant failure in the follow-up period. Apart from the admission for debridement and re-suturing in five patients, none had any history of re-admission for orthopaedic or other co-morbidities. Post-operative improvement of ROM, WOMAC score, revised Oxford knee score and knee alignment have been listed in Table 3.

**Table 1.** Comparison of demographic data of two contemporary high flexion knee designs.

	Mean age	Mean BMI	Gender	Side	Cases with FFD	Co-morbidities
Depuy (n = 100)	65.5 (r = 51–79)	29.4 (r = 22.5–32.4)	M = 22	L = 51	57	Hypertension-72%
			F = 78	R = 49		Type 2 DM-21%
						Hypothyroidism-11%
						Dyslipidaemia-10%
						Ischaemic heart ds-4%
						Asthma-1%
						Depression-1%
						None-22%
Zimmer (n = 100)	63.7 (r = 52–85)	29.7 (r = 21.8–31.6)	M = 13	L = 37	55	Hypertension-70%
			F = 87	R = 63		Hypothyroidism-13%
						Type 2 DM-13%
						Dyslipidaemia-8%
						Ischaemic heart ds-7%
						Asthma-2%
						Depression-2%
						None-28%

M = male, F = female, L = left, R = right, r = range, FFD = fixed flexion deformity.

**Table 2.** Enumeration of implant sizes used of two contemporary high flexion knee designs.

	Depuy (n = 100)	Zimmer (n = 100)
Femoral component size	2.5–32%	D – 51%
	2–28%	C – 29%
	3–32%	E – 10%
	1.5–1%	F – 10%
	4–6%	
	3.5–1%	
Tibial component size	3–45%	3–37%
	2.5–23%	4–32%
	2–22%	5–19%
	4–5%	6–10%
	5–4%	2–2%
	3.5–1%	
Insert size	10–60%	10–71%
	12.5–22%	12–27%
	8–18%	14–2%

**Table 3.** Comparison of functional outcome in the two contemporary high flexion knee designs.

		Mean ROM	FFD	Mean WOMAC score	Mean modified Oxford score	Knee alignment			
						Varus	Normal	Valgus	Mean
Depuy	Pre-op	94.6° (r = 60–150°)	57% (m = 4° r = 5–20°)	63 (r = 55–73)	13 (r = 8–19)	62%	38%	0	6° varus (r = 5° valgus–30° varus)
	Post-op	134.6° (r = 110–145°)	0	3.5 (r = 1–8)	45.6 (r = 34–48)	0	100%	0	5.3° valgus (r = 4° –10° valgus)
Zimmer	Pre-op	95.2° (r = 50–140°)	55% (m = 4.25° r = 5–30°)	63.5 (r = 55–73)	12.7 (r = 8–19)	74%	26%	0	7.6° varus (r = 10° valgus–40° varus)
	Post-op	133.4° (r = 115–145°)	0	4.65 (r = 1–8)	39.9 (r = 32–48)	0	100%	0	5.2° valgus (r = 4° –12° valgus)

Pre-op = pre-operative, Post-op = post-operative, ROM = range of motion, FFD = fixed flexion deformity, m = mean, r = range.

In the Depuy group, mean knee alignment was 5.3° valgus. The femoral component was satisfactorily positioned in 98%. Femoral notching was noted in 2% and there was no medio-lateral component overhang. Tibial component position was satisfactory in 95% with posterior overhang noted in 2% and medial overhang in 1%. The tibial stem was directed centrally in both antero-posterior and lateral views in 98% cases. In 2% cases, it was directed posteriorly. There was no patellar subluxation/dislocation. None had osteolysis or aseptic loosening at the two year follow-up.

In the Zimmer group, mean knee alignment was 5.2° valgus. The femoral component was satisfactorily positioned in 97%. Femoral notching was noted in 1% while excess femoral component flexion was noted in 2%. Tibial component position was satisfactory in 97% cases. There was no overhang but the tibial stem was directed postero-laterally in 2% and posteriorly in 1%. There was no patellar subluxation/dislocation. There was no sign of osteolysis or aseptic loosening at the two year follow-up.

## Discussion

**Figure 1.**
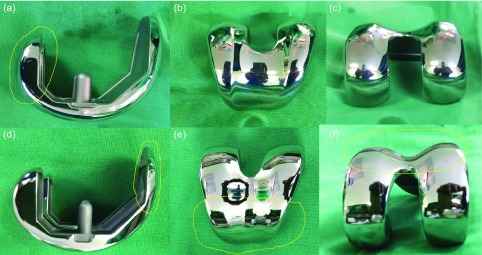
(a) Lateral view of Depuy CR 150 showing extended posterior condyle (sigma “J” curve), (b) superior view of Depuy CR 150, (c) posterior view of Depuy CR 150, (d) lateral view of Zimmer high flex showing decreased anterior flange thickness, (e) superior view of Zimmer high flex showing decreased anterior flange width, (f) posterior view of Zimmer high flex showing increased trochlear groove angle.

Patients have conventionally used pain relief and amount of flexion achieved as valuable indices of satisfaction after total knee replacement (TKR). Deep knee flexion is required in some parts of the world especially in Asian countries for cultural and religious reasons. Stair climbing requires 90–120° of flexion [6], using commode requires about 135° and activities like squatting, sitting cross legged or kneeling require about 165° of flexion [7]. Activities, such as meditation, yoga, gardening or playing golf which are few of the many activities enjoyed by potential patients for TKR, often require knee flexion greater than 150° [6–8]. Hence design-related modifications, to allow high flexion in a biomechanically safer environment, have been brought in by several companies [9]. There are various factors affecting the range of motion. Female gender, higher body mass index, pre-operative low range of motion [3], associated co-morbidities hampering mobility [8], component malposition, improper patello-femoral tracking, overstuffed patello-femoral joint, inadequate flexion gap and inadequate posterior femoral osteophyte removal are associated with decreased post-operative achievable flexion [10–14]. On the other hand, various prosthetic designs have been implemented to improve flexion. Depuy Sigma CR 150 system and Zimmer NexGen High Flex Knee system are the two popular prosthetic knee designs used in our setup which claim to accommodate high flexion up to 150° with adequate safety and reduced chances of edge loading. The Depuy system (Figure 1a) has an extended posterior condylar curve (sigma “J” curve) and decreased posterior condylar radii to improve posterior femoral rollback and hence flexion. On the other hand, the Zimmer femoral component incorporates decreased anterior flange thickness (Figure 1d) and width (Figure 1e) with increased trochlear groove angle (Figure 1f) to prevent overstuffing of the patello-femoral joint along with decreased condylar radii and thus improve the range of motion.

In our study, we have compared the two year follow-up results of total knee replacement with Sigma CR150 High Flex Knee prosthesis (DePuy, Warsaw, Indiana) and NexGen High Flex Knee prosthesis (Zimmer, Warsaw, Indiana). The mean ROM increased from 94.6° to 134.6° after TKR in Depuy group, which was statistically significant (*p* = 0.000). The mean ROM increased from 95.2° to 133.4° after TKR in Zimmer group, which was also statistically significant (*p* = 0.000). The results are consistent with those of Han et al. [15] where the two years post-operative ROM was 131.0 ± 10.5°. The ROM achieved in Depuy group was greater than in Zimmer group but it was statistically not significant (*p* = 0.46). The mean WOMAC score improved from 63 pre-operative to 3.5 at two years post-operative in Depuy group which was statistically significant (*p* = 0.00). The mean WOMAC score also improved from 63.5 pre-operative to 4.65 at two years post operative in the Zimmer group which was statistically significant (*p* = 0.00). A difference of 1.15 points was noted between the two years post-operative WOMAC score in Depuy and Zimmer groups, which was statistically significant (*p* = 0.00) but clinically insignificant (minimal clinically important difference for WOMAC score is 15) [16]. The mean modified Oxford knee score was found to improve statistically significantly in Depuy group from 13 pre-operative to 45.6 post-operative (*p* = 0.00) and in Zimmer group from 12.7 pre-operative to 39.9 post-operative (*p* = 0.00). A difference of 5.7 points was noted between the two years post-operative modified Oxford score in Depuy and Zimmer groups, which was statistically (*p* = 0.00) as well as clinically significant (minimal clinically important difference of Oxford knee score is five points) [17]. Hence functionally results in Depuy group were better than Zimmer.

Radiological results were comparable in both groups as there was no sign of osteolysis, mal-alignment of limb or implant failures at the two year follow-up.

Thus, we conclude that the Depuy group fared better than the Zimmer group in terms of functional outcome. However, it is very difficult to rate one design above the other based on our small sample size and short duration of follow-up. This study lays a basic structure for further research in the same direction with a larger sample size and longer duration of follow-up.

## Conflict of interest

The authors declare no conflict of interest in relation with this paper.
